# Variation in Minerals, Phenolics and Antioxidant Activity of Peel and Pulp of Different Varieties of Peach (*Prunus persica* L.) Fruit from Pakistan

**DOI:** 10.3390/molecules17066491

**Published:** 2012-05-30

**Authors:** Maleeha Manzoor, Farooq Anwar, Zahed Mahmood, Umer Rashid, Muhammad Ashraf

**Affiliations:** 1 Department of Chemistry and Biochemistry, University of Agriculture, Faisalabab 38040, Pakistan; Email: maleehamanzoor2008@yahoo.com (M.M.); drzahiduaf2003@yahoo.com (Z.M.); 2 Department of Chemistry, University of Sargodha, Sargodha 40100, Pakistan; 3 Institute of Advanced Technology, Universiti Putra Malaysia, Serdang 43400, Selangor, Malaysia; 4 Department of Botany, University of Agriculture, Faisalabad 38040, Pakistan; Email: ashrafbot@yahoo.com

**Keywords:** fruits, antioxidant compounds, Folin reagent, radical scavenger, reducing power

## Abstract

Peach (*Prunus persica* L.), being a potential source of bioactive compounds, has been demonstrated to have medicinal benefits. In this study variation of minerals and antioxidant characteristics (total phenolic contents, total flavonoid contents, reducing power, inhibition of peroxidation using linoleic acid system and DPPH free radical scavenging activity) between peel and pulp parts of different peach varieties, namely Golden, Shireen, and Shahpasand were investigated. The peel and pulp extracts, derived from the varieties analyzed, exhibited an appreciable amount of total phenolics (TP) and total flavonoids (TF), ranging from 1,209.3–1,354.5, 711.7–881.3 mg GAE/100 g and 599.7–785.5, 301.3–499.7 mg CE/100 g on a dry weight basis, respectively. Reducing power of peel and pulp extracts (12.5 mg/mL concentration) ranged from 2.57–2.77 and 1.54–1.99. The inhibition of linoleic acid peroxidation and DPPH scavenging activity of the extracts varied from 70.8–80.9% and 66.8–76.5% in peels, and 51.9–60.1% and 43.4–49.1% in pulps. The mineral analysis revealed that the content of K was highest in both parts of the peach fruit followed by Mg, Ca, Fe, Mn and Zn. The results of our present study indicate that peach peel had significantly higher levels of minerals, antioxidant capacity and phenolics than those of the pulp, suggesting the intake of unpeeled peach as a potential source of high-value components. The peach peel can be a useful as a viable source of natural antioxidants for functional foods and nutraceutical applications.

## 1. Introduction

Fruits have long been regarded as a valuable food commodity with potential health benefits, due in part to their natural antioxidant components, which can contribute to decreasing the incidence of cardiovascular and other chronic diseases [1,2]. It has been revealed that carotenoids and polyphenols such as phenolics, flavonoids, anthocyanins, and phenylpropanoids present in fruits might act as antioxidants or as agents with other therapeutic properties contributing to cardio protective action [3].

Peach (*Prunus persica* L.) fruits have high economic and nutritional value [4,5]. Carbohydrates, organic acids, minerals and dietary fiber are among the major constituents of peach fruit, which contribute to the nutritional quality of both fresh fruits and the juices [6]. Fully ripened peach fruits, mostly having golden yellowish flesh, are usually sweeter because they exhibit lower acidity. On the other hand, fruits with yellow flesh normally have an acidic flavor together with sweet taste. A peach is exceptionally rich in vitamin A and potassium, in addition to having considerable amounts of other valuable components such as organic acids and natural sugars, *etc*. These constituents certainly elevate the nutritional status of the peach fruit. With regard to medicinal functions, dietary intake of peach can reduce the generation of ROS (reactive oxygen species) in human blood plasma and provide protection from a number of chronic diseases [7]. Peach fruits have laxative properties and are appropriate to prevent constipation and for the treatment of duodenum ulcers. Phenolic acids, flavonoids, and anthocyanin compounds serve as a major source of potential antioxidants in peach fruit, which might have been responsible for these medicinal functions [2,8,9]. The phytochemical contents of fruits are influenced by numerous factors such as climatic conditions, agronomic practices, and varietal differences [10]. Moreover, contents of organic acids, carbohydrates and phenolics are not uniformly distributed within different parts of fruits, and most of them are concentrated in the epidermal and sub-epidermal layers of fruit [11,12].

The commercial and domestic uses of large quantity of fruits, especially for the purposes of juice, and/or processed sauces and slice production [13], result in the generation of large quantity of seeds and peel as agro-wastes. Many published reports reveal that peel of various fruits *i.e*., grape, citrus, pomegranate, mango, *etc*. contain higher amount of phenolics and flavonoids as compared to their flesh parts [14,15,16,17]. For example, Manzoor *et al*. [12] reported that apple fruit peels have higher antioxidant activity compared to the pulp extract. Gorinstein *et al*. [14], showed that phenolics and mineral in persimmons and apple peel have higher activity compared to the pulp. Leontowicz *et al.* [15] reported that apple and pear peels have higher antioxidant contents compared to the pulps.

Currently, significant efforts have been directed on plants and their by-products to extract natural and low-cost antioxidants that can replace synthetic additives [18,19]. In Pakistan peach are mainly grown in Khyber Pukhtunkha and Baluchistan, however some low chill and early maturing cultivars are also distributed in Pothwar area of Punjab. Peach is a traditional food crop of the northern areas of Pakistan, occupying an estimated area of 4,543 hectares yielding some 48,284 tonnes. Kalat, Quettea, Peshawar, Swat valley and some regions of Kohistan hills are the major peach growing areas. In Swat (Khyber Pukhtunkha) and Baluchistan, Golden, Shahpasand and Shireen are the most commonly grown varieties of peach fruit and have high market value/potential [20]. The available information on the minerals and antioxidant properties of flesh and peels of peach fruit is sporadic. Also, to the best of our understanding, no previous studies have documented the antioxidant activity of different varieties of peach commonly cultivated in Pakistan, so the main objective of this investigation was to evaluate the mineral profile, phenolic and flavonoid contents and antioxidant attributes of pulp and peel parts of three varieties of peach locally grown in Pakistan. 

## 2. Results and Discussion

### 2.1. Extract Yields, Total Phenolics and Total Flavonoids Content

Peel and pulp from the fruits of three different peach varieties offered promising amounts of extractable matter with aqueous methanol (80% methanol), as shown in Table 1. The extraction yield of antioxidant components varied from 14.1–18.9 g/100 g for peel and 7.4–10.3 g/100 g for pulp, showing significant variation between the two parts tested. A relatively higher extraction yield was obtained for peel compared to that of the pulp of the tested peach fruits. Polar solvents such as methanol, ethanol, ethyl acetate and acetone, *etc*. are widely used for the extraction of antioxidant components from plant materials; however, extraction with methanol, especially aqueous methanol, often results in a higher recovery of total extractable compounds. The significant differences (*p* < 0.05) in the extraction yield between different parts of same fruit might be ascribed to the varying availability of extractable components, resulting from the varied chemical composition of different tissues (peel and pulp) used [12]. 

The total phenolic contents were quantified using the Folin-Ciocalteau method, which relies on the transfer of electrons from phenolic compounds to the Folin–Ciocalteu reagent in an alkaline medium thereby producing a blue complex, the intensity of which is measured spectrophotometrically against mg GAE/100 g of DW as a standard [21,22,23].

Total phenolics of pulp and peel of the three different peach fruit varieties (Golden, Shireen and Shahpasand) varied significantly (*p* < 0.05) between the two parts tested (Table 1). The amount of total phenolics as determined in the present investigation in the three different varieties of peach ranged from 711.7–881.3 mg GAE/100 g of DW for pulp extracts, and 1,299.3–1,354.5 mg GAE/100 g of DW for peel extract. Among different peach varieties tested, the peel and pulp of cv. Golden exhibited the highest phenolic contents (1354.5 and 881.3 mg GAE/100 g of DW), whereas these amounts were lowest (711.7 and 1,299.3 mg GAE/100 g of DW) in Shahpasand. The total phenolic contents as investigated in the present analysis of peach were found to be lower than those reported in other Rosaceae fruits *i.e.*, berries (12.4–50.8 mg/g GAE) [24] and apple peel (1.907.5–2.587.9 mg GAE/100 g DW) and apple pulp (1.185.2–1.475.5 mg GAE/100 g DW) extracts [12]. In support of our present findings, a study by Chang *et al.* [14] also revealed that peels of peach had 2–2.5 times higher amounts of phenolics than those detected in flesh and whole fruit extracts. According to the above referred study [13], the contents of total phenols were 467 to 801 mg/kg in flesh extracts, 415 to 765 mg/kg in whole fruit extracts, and 877 to 1896 mg/kg in peel extracts.

Total flavonoids (TF) of pulp and peel of the three different varieties of peach fruit also varied considerably (Table 1). In the peel extract, total flavonoids ranged from 599.7–785.5 mg CE/100 g DW, whereas, this amount decreased in the pulp extract to levels of 301.3–499.7 mg CE/100 g DW. The peel of Golden peach exhibited significantly (*p* < 0.05) higher content of flavonoids (785.5 mg CE/100 g) compared with those of Shireen and Shahpasand (677.4 and 599.7 mg CE/100 g, respectively). In case of pulp extract, cv. Shireen had higher contents of TF (499.7 mg CE/100 g) whereas no significant variation was observed between Golden (383.7 mg CE/100 g) and Shahpasand (301.3 mg CE/100 g). Overall, var. Golden exhibited higher contents of phenolics and flavonoids than the others.

**Table 1:** Percent extraction yield, total phenolic contents (TPC) and total flavonoid contents (TFC) of peel and pulp extracts from different varieties of peach (*Prunus persica* L.) fruit.

Variety	Dry matter (%)		Extract Yield (%)		TPC (mg gallic acid equivalent/100 g dry weight)		TFC (mg catechin equivalent/100 g dry weight)	
	Peel	Pulp	Peel	Pulp	Peel	Pulp	Peel	Pulp
Golden	29.9 ± 0.6	18.3 ± 0.4	18.9 ± 0.4	10.3 ± 0.2	1354.5 ± 18.6	881.3 ± 12.3	785.5 ± 15.3	383.7 ± 7.2
Shireen	30.7 ± 0.5	16.6 ± 0.4	15.8 ± 0.3	9.1 ± 0.2	1300.9 ± 17.5	781.8 ± 11.5	677.4 ± 13.5	499.7 ± 9.4
Shahpasand	34.3 ± 0.7	21.1 ± 0.4	14.1 ± 0.3	7.4 ± 0.2	1209.3 ± 22.7	711.7 ± 14.9	599.7 ± 11.7	301.3 ± 7.7
**Mean**	31.6 ± 0.6 ^a^	18.6 ± 0.4 ^b^	16.3 ± 0.4 ^a^	8.9 ± 0.2 ^b^	1288.4 ± 25.9 ^a^	791.6 ± 15.8 ^b^	687.5 ± 13.3 ^a^	394.9 ± 7.9 ^b^

Data are mean ± SD (*n* =3 × 3, *P* < 0.05); Different superscript alphabets within the Mean’s row indicate significant differences (*P* < 0.05) between peel and pulp.

The contents of TP and TF as determined in the peach peels were found to be higher than those of the corresponding pulp. This was due to the fact that phenolic substances generally accumulate in higher concentration in the outer tissues of plant parts such as fruits, seeds, and barks due to their prospective function in protection against sun-derived ultraviolet (UV) radiation. They also act as protectors against pathogen and pest attack [25]. Cevallos-Casals *et al*. [9] also reported that contents of phenolic compounds vary within different tissues of same fruits and are mostly concentrated in the epidermal and sub-epidermal layers of the fruits. Higher concentrations of phenolics, carotenoids and ascorbic acid in peach peel compared to the pulp were also reported by other researchers [2,26].

### 2.2. Reducing Power of Peach Extract

The assessment of reducing power of a compound may act as a good indicator of its potential antioxidant activity. Measurement of reducing power is mostly linked with the occurrence of reductants such as phenolics, which exert antioxidant action through donating their hydrogen atom or by breaking free radical chains. The presence of reducing agents in a typical sample causes the reduction of Fe^3+^ to Fe^2+^ and thus reductive capability can be monitored colorimetrically by monitoring the formation of Perl’s Prussian blue complex at 700 nm [27,28]. Table 2 presents the reductive capability of peel and pulp extracts of different varieties of peach fruit. The reducing potential of the tested extracts was recorded over a concentration range of 2.5 to 10.0 mg/mL. An almost general increase in activity with an increase in concentration was observed. Relatively, the extracts from peel showed significantly (*p* < 0.05) higher reductive capability than did the pulp extracts.

**Table 2:** Reducing power (absorbance values at 700 nm) of peel and pulp extracts from different varieties of peach (*Prunus persica* L.) fruit.

Variety	Conc. (mg/mL)	Pulp	Peel
Golden	0.0	0.060 ± 0.005	0.090 ± 0.008
	2.5	0.34 ± 0.01	0.59 ± 0.04
	5.0	0.61 ± 0.02	0.74 ± 0.03
	7.5	0.95 ± 0.01	1.19 ± 0.02
	10.0	1.12 ± 0.04	1.87 ± 0.05
	12.5	1.99 ± 0.02	2.77 ± 0.04
**Mean**		0.85 ± 0.02 ^a^	1.20 ± 0.07 ^b^
Shireen	0.0	0.040 ± 0.003	0.050 ± 0.004
	2.5	0.31 ± 0.01	0.52 ± 0.05
	5.0	0.58 ± 0.03	0.85 ± 0.04
	7.5	0.84 ± 0.04	1.10 ± 0.07
	10.0	1.09 ± 0.06	1.66 ± 0.06
	12.5	1.87 ± 0.05	2.57 ± 0.08
**Mean**		0.73 ± 0.01 ^a^	1.12 ± 0.05 ^b^
Shahpasand	0.0	0.030 ± 0.002	0.070 ± 0.009
	2.5	0.29 ± 0.02	0.57 ± 0.03
	5.0	0.48 ± 0.03	0.82 ± 0.05
	7.5	0.79 ± 0.05	1.17 ± 0.04
	10.0	0.99 ± 0.04	1.74 ± 0.05
	12.5	1.54 ± 0.03	2.66 ± 0.09
**Mean**		0.68 ± 0.01 ^a^	1.17 ± 0.03 ^b^

Data are mean (*n* = 3) SD ± (*n* = 3, *p* < 0.05); Different superscript letters within the Mean’s rows indicate significant differences (*p* < 0.05) between peel and pulp.

The reductive capability of peel and pulp extracts at 12.5 mg/mL, ranged from 2.57–2.77 and 1.54–1.99, respectively. In case of peel extracts, the highest (2.77) reducing power was exhibited by cv. Golden and the lowest (2.57) by cv. Shireen. For the pulp extracts, cv. Golden exhibited highest (1.99) reductive capability followed by Shireen (1.87) and Shahpasand (1.54). The mean reductive capability of the peel extract of the three varieties was significantly (*p* < 0.05) higher compared to that of the pulp extracts.

### 2.3. DPPH Radical Scavenging Activity

DPPH is a stable free radical with a deep violet color that offers an absorption maximum at a wavelength of 515 nm. In this test, the violet color of DPPH is reduced to a pale yellow color due to the abstraction of hydrogen atom from antioxidants. Assessment of the ability of an antioxidant or a plant extract to scavenging stable DPPH radical can be employed as a reliable measure to evaluate the antioxidant potential of samples [29]. A higher magnitude of DPPH radical scavenging is generally related to the superior antioxidant activity of the related sample extract [30]. 

**Figure 1:**
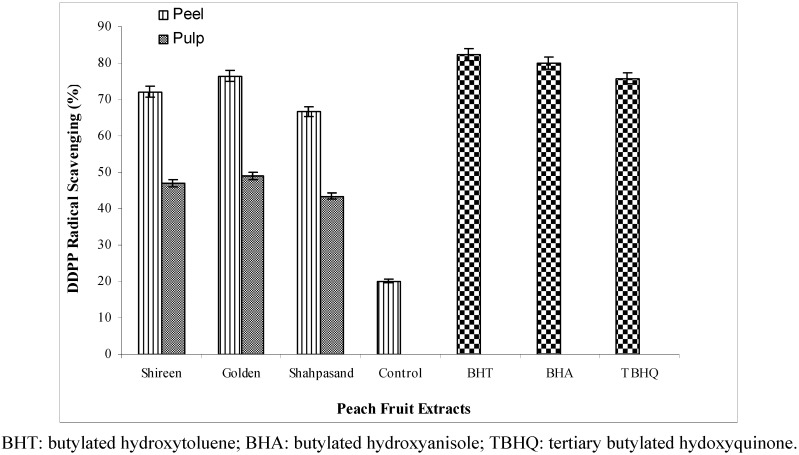
DPPH radical scavenging activity of 80% methanolic extracts from different varieties of peach (*Prunus persica* L.) fruit.

The DPPH radical scavenging activity of 80% methanolic extracts of peel and pulp was tested and compared with BHT, BHA and TBHQ (Figure 1). BHT and BHA and TBHQ acted as positive controls and gave the fastest color change from purple to yellow. In this assay the absorbance was measured at different intervals (1 to 12 min) from the beginning of the reaction. Maximum difference in scavenging activity was recorded at 5th min of the reaction and thus this was used for calculations. 

Peel extracts from all the peach cultivars exhibited appreciably higher scavenging activity, ranging from 66.8–76.5% compared to those of pulp extracts, 43.4–49.1% (Figure 1). The peel extract from Golden exhibited the highest (76.5%) scavenging activity, whereas the lowest (66.8%) was for Shahpasand. In case of pulp extracts, the highest (49.1%) and lowest (43.4%) scavenging capacities were recorded for Golden and Shahpasand, respectively. The scavenging activity of the positive controls BHT (82.2%) and BHA (80.1%) was higher compared to different peel and pulp extracts, however TBHQ (75.7%) exhibited scavenging potential comparable with a typical *var*. Golden peel. Analysis of variance (ANOVA) revealed no significant variation (*p* > 0.05) in radical scavenging capacity among peach varieties tested, however, the difference was significant (*p* < 0.05) between peel and pulp parts of each variety. 

**Figure 2:**
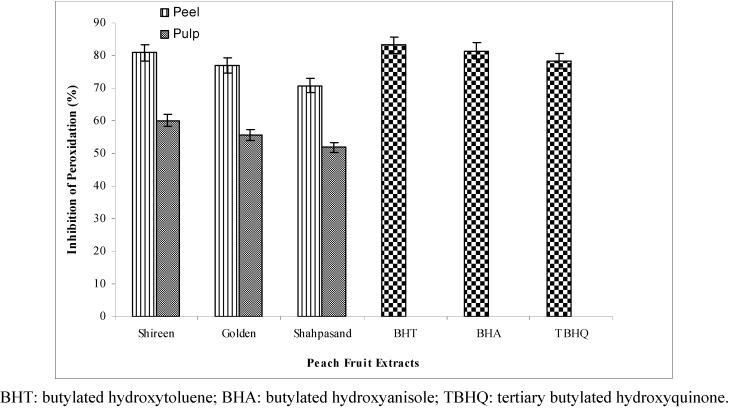
Antioxidant activity (in terms of percent inhibition of linoleic acid peroxidation) of 80% methanolic extracts from different varieties of peach (*Prunus persica* L.) fruit.

### 2.4. Antioxidant Activity of Peach Peel and Pulp Extracts in Linoleic Acid Peroxidation System

The inhibition of the linoleic acid peroxidation system was also used to assess the antioxidant activity of different varieties of peach fruit extracts. Both parts (peel and pulp) of peach fruit exhibited appreciable inhibition in the linoleic acid peroxidation system test, as shown in Figure 2. 

For peel extracts the inhibition of linoleic acid peroxidation ranged from 70.8% to 80.9%, whereas it was 51.9% to 60.1% in the case of pulp extracts. The peel and pulp extracts of Shireen (80.9% and 60.1%) were found to be more (*p* < 0.05) effective in inhibition of peroxidation, whereas, the lowest value was exhibited by Shahpasand (70.8% and 51.9 %). A significant variation (*p* < 0.05) between peel and pulp extract was observed, however, variation among different varieties was found to be non-significant. The inhibition values for the synthetic antioxidants (BHT, BHA and TBHQ), used as positive controls, were considerably higher than the pulp extracts while these were closely resembled with those of the tested peel extracts.

### 2.5. Minerals

The contents of minerals in peel and pulp of three different varieties of peach fruit are listed in Table 3. In this study significant differences (*p* < 0.05) were observed for each individual mineral between peel and pulp of fruit. Potassium (K) was the major macro-element of peach fruit. The contents of K and Ca in peach peel and pulp ranged from 1,240.4–1,330.1 and 1,520.2–1,690.4 mg/100 g DW and 76.6–87.5 and 40.8–59.7 mg/100 g DW, respectively.

**Table 3:** Mineral contents in peel and pulp of different varieties of peach (*Prunus persica* L.) fruit.

Mineral Contents (mg/100 g)	Variety					
	Shireen		Golden		Shahpasand	
	Peel	Pulp	Peel	Pulp	Peel	Pulp
K	1240.4 ± 24.8	1520.2 ± 31.5	1330.1 ± 26.4	1690.4 ± 33.1	1290.1 ± 23.9	1580.3 ± 30.2
Ca	87.50 ± 1.74	59.70 ± 1.33	81.70 ± 1.57	49.90 ± 1.11	76.60 ± 1.46	40.80 ± 0.83
Mg	92.70 ± 1.81	61.50 ± 1.24	100.9 ± 0.2	69.30 ± 1.39	85.50 ± 1.54	54.30 ± 1.13
Mn	0.69 ± 0.2	0.47 ± 0.03	0.74 ± 0.1	0.53 ± 0.04	0.70 ± 0.03	0.41 ± 0.02
Fe	7.01 ± 0.14	1.62 ± 0.01	6.53 ± 2.7	1.89 ± 0.03	5.14 ± 1.12	1.59 ± 0.04
Zn	0.59 ± 0.03	0.35 ± 0.02	0.51 ± 0.05	0.30 ± 0.02	0.40 ± 0.03	0.29 ± 0.02
**Mean**	238.14 ± 4.76 ^a^	273.97 ± 4.35 ^b^	253.41 ± 5.03 ^a^	302.05 ± 6.01 ^b^	239.78 ± 4.99 ^a^	279.61 ± 5.61 ^b^

Data are mean (*n* = 3) SD ± (*n* = 3, *p* < 0.05); Different superscript letters within the Mean’s row indicate significant differences (*p* < 0.05) between peel and pulp.

The peel and pulp of Golden exhibited the highest contents of K (1,330.1 and 1,690.4 mg/100 g), whereas the lowest (1,240.4 and 1,520.2 mg/100 g) was found in Shireen, respectively. The concentration of Ca was highest in peel (87.5 mg/100 g) and pulp (59.7 mg/100 g) of Shireen, and lowest in peel (76.6 mg/100 g) and pulp (40.8 mg/100 g) of cv. Shahpasand.

Potassium as an electrolytic mineral, which plays an important role in ionic balance and it contributes to maintenance of cell organization and permeability [31]. Calcium is known in human nutrition for the development and growth of skeletal e.g. bones and teeth, as well as coenzymes in the metabolic regulations of biomolecules [32].

The content of Mg and Mn in case of peel was within 85.5–100.9 and 0.69–0.74 mg/100 g whereas for pulp it varied from 54.3–69.3 and 0.41–0.53 mg/100 g, respectively. Magnesium plays an important role in nervous system stability, muscle contraction, as an activator of alkaline phosphatase and it can also be used as an alternative to calcium in the body [33]. Manganese is one of the important essential elements but it is only required in trace amounts for maintaining proper carbohydrates metabolism as well as an antioxidant in superoxide dimutase enzymes [31].

The content of Fe and Zn for peach peel ranged from 5.14–7.01 and 0.40–0.59 mg/100 g whereas it was 1.59–1.89 and 0.29–0.35 mg/100 g, in the case of pulp, respectively. The contents of Fe and Zn were highest in peel and pulp of Shireen (7.01 and 0.589 mg/100 g) while Shahpasand exhibited the lowest (5.14 and 0.401 mg/100 g) concentration of these two essential elements.

Iron is an important trace element and is a core of red blood cells. Its deficiency can cause anemia [31]. Zinc is reported as a coenzyme for over 200 enzymes involved in immunity, new cell growth, acid base regulation etc. Lack of sufficient amounts of Zn may result in reduced activity of related enzymes e.g., carbonic anhydrase [34].The present mineral analysis results were in agreement with those of Basar [35] who reported that potassium (K) was the most abundant nutrient in fruits of different peach varieties, followed by magnesium (Mg), calcium (Ca), nitrogen (N), iron (Fe), zinc (Zn) and manganese (Mn).

### 2.6. Correlations among the Results of Different Antioxidant Assays

As far as the correlation among results of different antioxidant assays is concerned a very good correlation between TPC and TFC was observed for peel (0.970***) of different peach varieties (Table 4). Manzoor *et al*. [12] also reported a good correlation in peel of five different cultivars of apple fruit from Pakistan. Similarly, a strong correlation between total phenolics and flavonoids contents in selected fruits and vegetable was also reported by Lin and Tang [36]. Both phenolic and flavonoids components having strong redox potential and act as potent antioxidants. Conversely, a weak correlation was found among TPC and TFC of pulps from different varieties of peach (0.320^ns^) that might be in due part to the varietal variation of these compounds in pulps.

**Table 4:** Comparison of results from different antioxidant assays as represented by correlation coefficients.

	Variable	TPC	TFC	% inhibition	DPPH	Reducing power
**Peel**	**TPC**	1				
	**TFC**	0.970 ***	1			
	**% inhibition**	0.709 *	0.517^ns^	1		
	**DPPH**	0.995 ***	0.989 ***	0.638 *	1	
	**Reducing power**	0.418^ ns^	0.625 *	−0.343 ^ns^	0.503^ ns^	1
**Pulp**	**TPC**	1				
	**TFC**	0.320 ^ns^	1			
	**% inhibition**	0.385^ ns^	0.997 ***	1		
	**DPPH**	0.973 ***	0.528^ ns^	0.586^ ns^	1	
	**Reducing power**	0.934 ***	0.636 *	0.687 *	0.991 ***	1

ns: non significant; *: level of significance.

For peel extract, a good correlation between TFC and DPPH (0.989***) was noted, while a weak correlation of TFC with reducing power (0.625*) was observed. This correlation was also non- significant and weak (*r* = 0.528^ns^ and 0.636*) in the case of the pulp extracts. A good correlation in case of peel extracts compared to the pulp might be attributed to the presence of higher amounts of flavonoids and phenolics in the peel extracts from the different varieties tested.

A positive correlation between DPPH and TPC was observed both for peel (0.995***) and pulp (0.973***) extracts of different peach varieties. However, the correlation between DPPH and TFC in the case of peel (0.989***) extracts was very strong, but non-significant for pulp (0.528^ns^) extracts. Meanwhile, a weak correlation between percent inhibition and TPC for peel (0.709*) extracts and a non-significant correlation in case of pulp (0.385^ns^) extracts was also observed. Both for peel and pulp extracts, a weak and non-significant correlation between percent inhibition and DPPH (0.638* and 0.586^ns^) and reducing power (0.343^ns^ and 0.687*) were observed. This weak correlation found between the present results of percent inhibition with those of reducing power and DPPH assays might be linked to the fact that some antioxidant components show their antioxidant potential not only by acting as hydrogen donors, but also as oxygen scavengers [12]. 

## 3. Experimental

### 3.1. Samples

Fully ripened (golden yellow colored) fresh fruits of three varieties of peach (*Prunus persica* L.), namely Golden, Shireen and Shahpasand, were collected from the vicinity of Swat (Khyber Pukhtunkha) and Quetta (Baluchistan), Pakistan during the summer of 2010 (May to July 2010). Three different samples of each variety (each sample derived from at least ten to fifteen trees in each locality) were randomly harvested. The fruits were further identified and authenticated from the Department of Horticulture, University of Agriculture, Faisalabad, Pakistan. 

### 3.2. Chemicals and Reagents

Linoleic acid, 2,2-diphenyl-1-picrylhydrazyl (DPPH) radical, Folin-Ciocalteu, butylated hydroxyl-anisole (BHA), butylated hydroxytoluene (BHT) and gallic acid were procured from Sigma Chemical Co. (St Louis, MO, USA). All other chemicals and reagents (analytical grade) used in the present investigation were from Merck (Darmstadt, Germany) or Sigma Aldrich (Buchs, Switzerland), unless stated otherwise.

### 3.3. Sample Preparation

After washing thoroughly with tap water, the fruits were peeled and the seed kernel was removed using a steel knife. The recovered pulp and peel portions were sliced into approx. 1 × 1 cm cubes.

### 3.4. Dry Matter Determination

Due to the varying levels of moisture in the peach fruits of the three varieties, all calculations were done on a dry matter basis. For dry matter determination, an AOAC procedure (method 925.10) was used [37]. Briefly, a known weight (5.0 g) of the sample was placed in an electric-oven (Memmert, Germany) for drying at 105 °C, until a constant weight achieved.

### 3.5. Antioxidant Activity of Fruits

#### 3.5.1. Extraction

Homogenized fruit from each variety (each 20 g) was extracted with 80% aqueous methanol (200 mL, methanol-water, 80:20, v/v) at room temperature for 8 h using an electric orbital shaker (Gallenkamp, London, UK). The residues and the extracts were separated by filtering through a filter paper; the residues obtained were re-extracted twice with afresh portion of extraction solvent. The extracts recovered from three extractions were combined and excess of the solvent distilled-off in a vacuum rotary evaporator (EYELA, Tokyo, Japan) at 45 °C. The semi-solid extracts obtained were quantitatively transferred to the extraction solvent and preserved at 4 °C, until used for further experiments. 

#### 3.5.2. Determination of Total Phenolics Content (TPC)

A colorimetric method, based on Folin-Ciocalteu reagent, was used to appraise the amount of total phenolics [38]. The process involved the mixing of crude extract (50 mg) with Folin-Ciocalteu reagent (0.5 mL) and deionized water (7.5 mL). After waiting for 10 min, 20% aqueous sodium carbonate (w/v, 1.5 mL) was added and then the mixture incubated at 40 °C in a water bath for 20 min, followed by cooling in an ice bath. The absorbance of the final mixture was monitored at 755 nm (U-2001 Spectrophotometer, Hitachi Instruments Inc., Tokyo, Japan). For calculation of the TP amount, a standard gallic acid calibration curve, prepared by running solutions in the concentration range of 10–200 mg/L(R^2^ = 0.9980), was constructed. The amounts of total phenolics were expressed as gallic acid equivalents (GAE) mg/100 g of dry matter. 

#### 3.5.3. Determination of Total Flavonoid Contents (TFC)

The amounts of TF were determined colorimetrically. A previously described method [39] was used wherein fruit extract (1mL containing 0.1 mg/mL dry matter) was mixed with water (4 mL) in a 10 mL volumetric flask. At the start, aqueous 5% NaNO_2_ (3 mL) was added to the volumetric flask, then at 5 min, 10% AlCl_3_ (0.3 mL) and at 6 min1.0 M NaOH (2 mL) were added sequentially. Finally, the volume was brought up to 10 mL by adding more distilled water. The reaction mixture was mixed thoroughly in the flask for homogenization. The absorbance was noted at 510 nm using a spectrophotometer. TFC, calculated using a standard calibration curve, were reported as catechin equivalents (mg CE/100 g of dry matter). 

#### 3.5.4. DPPH. Scavenging Assay

The 2,2′-diphenyl-1-picrylhydrazyl (DPPH) free radical scavenging capacity of the extracts was assessed following a previously described procedure [40]. In brief, freshly prepared DPPH methanolic solution (5.0 mL, 0.025 g/L) and the extract (1.0 mL, containing 25 µg/mL of dry matter in methanol) were mixed in a test tube. Absorbance of the reaction mixture was recorded at different time intervals, starting with 0 to 12 min at 515 nm. A Hitachi U-2001 spectrophotometer was used for recording the optical density. The remaining amounts of DPPH radical (DPPH**^.^**) were calculated from a standard calibration curve. Absorbance measured at 5th min was used for the comparison of the radical scavenging activity of the extracts. Butylated hydroxytoluene (BHT), butylated hydroxyanisole (BHA) and tertiary butylhydroquinone (TBHQ) were used as positive controls for comparison purposes.

#### 3.5.5. Determination of Antioxidant Activity in Linoleic Acid System

The antioxidant activity of the tested peach fruit extracts was also determined following the measurement of inhibition of linoleic acid peroxidation [41]. In this test, the fruit extract (5 mg of each peach variety) was mixed independently with an emulsion which contained solution of linoleic acid (0.13 mL), 99.8% ethanol (10 mL) and sodium phosphate buffer (pH 7, 10 mL, 0.2 M). The mixture was brought to 25 mL with distilled water and incubated at 40 °C up to 360 h. The magnitude of linoleic acid oxidation was measured by peroxide formation according to the thiocyanate method as described by Yen *et al.* [42]. A control, containing all reagents, except the fruit extracts, was also processed under similar conditions. Three synthetic antioxidant compounds, BHT, BHA, and TBHQ were used as positive controls for comparison purposes. Percent inhibition of linoleic acid oxidation was calculated by the expression:


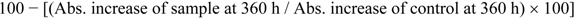


#### 3.5.6. Determination of Reducing Power

The reducing power of the fruit extracts was determined according to a previously described procedure [43] with slight modifications. The extracts (2.5–12.5 mg/mL) were mixed with sodium phosphate buffer (0.2 M, pH 6.6, 5.0 mL) and potassium ferricyanide (5.0 mL, 1.0%) in a test tube. The reaction mixture was placed in a water bath at 50 °C for 20 min and then 10% trichloroacetic acid (5 mL) was added followed by centrifugation of the mixture at 980 g for 10 min at 5 °C using a refrigerated centrifuge (CHM-17; Kokusan Denki, Tokyo, Japan). After centrifugation, the upper phase of the reaction mixture (*ca.* 5.0 mL) was collected and diluted further by adding distilled water (5.0 mL) and 0.1 % ferric chloride (FeCl_3_) solution (1 mL).The absorbance of the final solution was read at 700 nm using a spectrophotometer.

### 3.6. Minerals Composition

#### 3.6.1. Preparation of Samples for Mineral Analysis

Minerals analysis was performed spectrophotometrically and flame photometrically. Briefly, dried material of peel and pulp (1.0 g in either case) was taken in a digestion flask containing concentrated sulphuric acid (H_2_SO_4_) (5 mL). Sahito *et al*. [44] also processed a blank involving the addition of all reagents except the analyte/sample. The flasks were subjected to heating for about 60 min on an electric hot plate (HP 220, UTEC Products Inc., Albany, NY, USA) at 80 to 90 °C, then the temperature was increased to 150–160 °C, and the heating continued. In the mean time, an appropriate volume of HNO_3 _(concentrated) and 30% hydrogen peroxide (H_2_O_2_) were occasionally added into the flasks, and boiling/heating continued until clear solutions obtained indicating the complete digestion/oxidation of the organic matter. After that, the contents of the flasks were allowed to cool at room temperature, a small volume of distilled water was added, mixed well and the solution filtered through Whatman No. 42 (<0.45 μm Millipore) filter paper. Finally, the volume was made up to 25mL in a volumetric flask by adding distilled water.

#### 3.6.2. Preparation of Standards and Analysis of Samples

For quantitative measurements, standard solutions of the elements, namely potassium (K), magnesium (Mg), manganese (Mn), calcium (Ca), iron (Fe), and zinc (Zn) were prepared by successive dilution of the stock standard solutions (1,000 ppm) of the respective elements in 2NHNO_3_. Analysis of the samples for Ca, Mg, Mn, Fe and Zn was done on an atomic absorption spectrophotometer (A Analyst 300, Perkins Elmer, NY, USA) whereas K was determined on a flame photometer (Sherwood, UK). Standard calibration curves were constructed for each element individually using linear correlation by the least square method after running standard solutions. The blank reading was used to make necessary corrections during calculation of elemental concentrations.

### 3.7. Statistical Analysis

Three different fruit samples of each variety were randomly assayed. Each sample was analyzed individually in triplicate and data reported as mean (*n* = 3× 3 × 1) ± SD (*n* = 9). The data thus generated were subjected to analysis of variance (ANOVA) using Minitab 2000 Version 13.2 statistical software (Minitab Inc., State College, PA, USA).

## 4. Conclusions

In the present research work, the minerals, phenolic contents and antioxidant activity of different parts (peel and pulp) of three locally available varieties of peach fruit from Pakistan were evaluated. Among the three varieties analyzed, Golden variety exhibited higher antioxidant activity and total phenolic contents compared to the other two varieties, whereas, analysis of minerals revealed that var. Golden and Shireen have maximum amounts of minerals. Besides, the present results also revealed that peach peel exhibited higher antioxidant activity compared to that of the pulp, indicating that removal of peel from such fruits may induce significant nutrient losses. Therefore, the intake of fruits along with their peels can be more beneficial to maximize functional food value of such fruits. Future study to assess the intake of valuable minerals and antioxidants in peach fruits on a per serving basis is further recommended.

## References

[1] Isabelle M., Leea B.L., Lim M.T., Koh W.P., Huang D., Ong C.N. (2010). Antioxidant activity and profiles of common fruits in Singapore. Food Chem..

[2] Gil M., Tomas-Barberan F., Hess-Pierce B., Kader A. (2002). Antioxidant capacities, phenolic compounds, carotenoids, and vitamin A contents of nectarine, peach, and plum cultivars from California. J. Agric. Food Chem..

[3] Gorinstein S., Zachwiejab Z., Katricha E., Pawelzikc E., Haruenkitd R., Trakhtenberge S., Martin-Belloso O. (2004). Comparison of the contents of the main antioxidant compounds and the antioxidant activity of white grapefruit and his new hybrid. Lebensm.-Wiss. U.-Technol..

[4] Kurz C., Carle R., Schieber A. (2008). Characterisation of cell wall polysaccharide profiles of apricots (*Prunus armeniaca* L.), peaches (*Prunus persica* L.), and pumpkins (Cucurbita sp.) for the evaluation of fruit product authenticity. Food Chem..

[5] Wolfe K.L., Kang X., He X., Dong M., Zhang Q., Liu R.H. (2008). Cellular antioxidant activity of common fruits. J. Agric. Food Chem..

[6] Versari A., Castellari M., Parpinello G.P., Riponi C., Galassi S. (2002). Characterization of peach juices obtained from cultivars Redhaven, Suncrest and Maria Marta grown in Italy. Food Chem..

[7] Tsantili E., Shin Y., Nock J.F., Watkins C.B. (2010). Antioxidant concentrations during chilling injury development in peaches. Postharvest Biol. Technol..

[8] Rupasinghe V.H.P., Clegg S. (2007). Total antioxidant capacity, total phenolic content, mineral elements, and histamine concentrations in wine of different fruit sources. J. Food Comp. Anal..

[9] Cevallos-Casals B.A., Byrne D., Okie W.R., Cisneros-Zevallos L. (2006). Selecting new peach and plum genotypes rich in phenolic compounds and enhanced functional properties. Food Chem..

[10] Tavarini S., Innocenti E.D., Remorini D., Massai R., Guidi L. (2008). Antioxidant capacity, ascorbic acid, total phenols and carotenoids changes during harvest and after storage of Hayward kiwifruit. Food Chem..

[11] Mattila P., Hellström J., Törrönen R. (2006). Phenolic acids in berries, fruits, and beverages. J. Agric. Food Chem..

[12] Manzoor M., Anwar F., Saari N., Ashraf M. (2002). Variations of antioxidant characteristics and mineral contents in pulp and peel of different Apple (*Malus domestica* Borkh.) cultivars from Pakistan. Molecules.

[13] Chang S., Tan C., Frankel E., Barrett D. (2000). Low-density lipoprotein antioxidant activity of phenolic compounds and polyphenol oxidase activity in selected clingstone peach cultivars. J. Agric. Food Chem..

[14] Gorinstein S., Zachwieja Z., Folta M., Barton H., Piotrowicz J., Zemser M., Weisz M., Trakhtenberg S., Martın-Belloso O. (2001). Comparative contents of dietary fiber, total phenolics, and minerals in persimmons and apples. J. Agric. Food Chem..

[15] Leontowicz M., Gorinstein S., Leontowicz H., Krzeminski R., Lojek A., Katrich E., Ciz M., Martin-Belloso O., Soliva-Fortuny R., Haruenkit R. (2003). Apple and pear peel and pulp and their influence on plasma lipids and antioxidant potentials in rats fed cholesterol-containing diets. J. Agric. Food Chem..

[16] Abrosca B.D., Pacifico S., Cefarelli G., Mastellone C., Fiorentino A. (2007). Limoncella apple, an Italian apple cultivar: Phenolic and flavonoid contents and antioxidant activity. Food Chem..

[17] Lata B. (2007). Relationship between apple peel and the whole fruit antioxidant content: Year and Cultivar variation. J. Agric. Food Chem..

[18] Peschel W., Sanchez-Rabaneda F., Diekmann W., Plescher A., Gartzıa I., Jimenez D., Lamuela-Ravento R., Buxaderas S., Codina C. (2006). An industrial approach in the search of natural antioxidants from vegetable and fruit wastes. Food Chem..

[19] Khan M.K., Abert-Vian M., Sylvie A., Tixier F., Dangles O., Chemat F. (2010). Ultrasound-assisted extraction of polyphenols (flavanone glycosides) from orange (*Citrus sinensis* L.) peel. Food Chem..

[20] Pakissan.com Home Page. http://www.pakissan.com/english/allabout/orchards/peach.shtml.

[21] Cevallos-Casals B.A., Cisneros-Zevallos L. (2003). Stoichiometric and kinetic studies of phenolic antioxidants from Andean purple corn and red-fleshed sweet potato. J. Agric. Food Chem..

[22] Huang D., Ou B., Prior R.L. (2005). The chemistry behind antioxidants capacity assays. J. Agric. Food Chem..

[23] Kim H., Moon J.Y., Kim H., Lee D.-S., Cho M., Choi H.K., Kim Y.S., Mosaddik A., Cho S.K. (2010). Antioxidant and antiproliferative activities of mango (*Mangifera indica* L.) flesh and peel. Food Chem..

[24] Kahkonen M.P., Hopia A.I., Vuorela H.J., Rauha J.P., Pihlaja K.P., Kujala T.S., Heinonen M. (1999). Antioxidant activity of plant extracts containing phenolic compounds. J. Agric. Food Chem..

[25] Toor R.K., Savage G.P. (2005). Antioxidant activities in different fraction of tomato. Food Res. Int..

[26] Tomas-Barbenin F.A., Gil M.I., Cremin P., Waterhouse A.L., HessPierce B., Kader A.A. (2001). HPLC-DAD-ESIMS analysis of phenolic compounds in nectarines, peaches, and plums. J. Agric. Food Chem..

[27] Pan Y., He C., Wang H., Ji X., Wang K., Liu P. (2010). Antioxidant activity of microwave-assisted extract of *Buddleia officinalis* and its major active component. Food Chem..

[28] Prasad K.N., Yang B., Zhao M., Sun J., Wei X., Jiang Y. (2010). Effects of high pressure or ultrasonic treatment on extraction yield and antioxidant activity of pericarp tissues of longan fruit. J. Food Biochem..

[29] Lee S.E., Hwang H.J., Ha J.S., Jeong H.S., Kim J.H. (2003). Screening of medicinal plant extracts for antioxidant activity. Life Sci..

[30] Abd-Ghafar M.F., Prasad K.N., Weng K.K., Ismail A. (2011). Flavonoid, hesperidine, total phenolic contents and antioxidant activities from Citrus species. Afr. J. Biotech..

[31] Ismail F., Anjum M.R., Mamon A.N., Kazi T.G. (2011). Trace metal contents of vegetables and fruits of Hyderabad retail market. Pak. J. Nutr..

[32] Han S.T., Tang R., Anderson L.K., Woerner T.E., Pei Z.M.B. (2003). A cell surface receptor mediates extracellular Ca^2+^ sensing in guard cells. Nature.

[33] Cowan A. (2002). Structural and catalytic chemistry of magnesium-dependent enzymes. Biometals.

[34] Stephanie S. (2010). Trace elements. Curr. Anaesthesia Crit. Care.

[35] Basar H. (2006). Elemental composition of various peach cultivars. Sci. Hortic..

[36] Lin J.Y., Tang C.Y. (2007). Determination of total phenolic and flavonoid contents in selected fruits andvegetables, as well as their stimulatory effects on mouse splenocyte proliferation. Food Chem..

[37] Association of Official Analytical Chemists (AOAC) (1990). Official Methods of Analysis of the Association of Official Analytical Chemists.

[38] Singleton V.L., Rossi J.A. (1965). Colorimetry of total phenolics with phosphomolybdic-phosphotungstic acid reagents. Am. J. Enol. Vitic..

[39] Jia Z., Tang M., Wu J. (1999). The determination of flavonoid contents in mulberry and their screening effects on superoxide radicals. Food Chem..

[40] Brands-William W., Cuvelier M.E., Berset C. (1995). Use of a free radical method to evaluate antioxidant activity. Lebensm. Wiss. Technol./Food Sci. Technol..

[41] Osawa T., Namiki M. (1981). A novel type of antioxidant isolated from leaf wax of eucalyptus leaves. Agric. Biol. Chem..

[42] Yen G.C., Duh P.D., Chuang D.Y. (2000). Antioxidant activity of anthraquinones and anthrone. Food Chem..

[43] Oyaizu M. (1986). Studies on products of browning reaction prepared from glucosamine. Jpn. J. Nutr..

[44] Sahito A., Kazi T.G., Jakhrani M.A., Kazi G.H., Shar G.Q., Memon M.A. (2002). Elementa investigation of *Momordica charantia* Linn., and *Syziginm jambolana* Linn., using atomic absorption spectrophotometer. Nucleus.

